# Peripheral Inflammatory Biomarkers in Parkinson’s Disease: Clinical Correlations and Stratification

**DOI:** 10.1007/s10571-026-01708-8

**Published:** 2026-03-08

**Authors:** Guo-Yun Jiang, Fan Li, Jin-Hui Yin, Ling-Xiao Cao

**Affiliations:** 1 Department of Clinical Laboratory, Shandong Provincial Hospital affiliated to Shandong First Medical University, Jinan, 250021 Shandong China; 2 Department of Neurology, Cheeloo College of Medicine, The Second Qilu Hospital of Shandong University, Shandong University, Jinan, 250033 Shandong China; 3 China National Clinical Research Center for Neurological Diseases, Beijing Tiantan Hospital, Capital Medical University, Beijing, 100070 China; 4 Department of Neurology, Beijing Tiantan Hospital, Capital Medical University, Beijing, 100070 China

**Keywords:** Parkinson’s disease, Peripheral inflammatory biomarker, Neuroinflammation, Neutrophil-to-lymphocyte ratio

## Abstract

Growing evidence underscores neuroinflammation’s role in Parkinson’s disease (PD), with accumulating evidence suggesting a potential role for peripheral inflammation. The clinical applicability and mechanistic relevance of peripheral inflammatory biomarkers in PD remain to be fully elucidated. We analyzed data from the Parkinson’s Progression Markers Initiative (PPMI), including longitudinal clinical assessments, blood counts, cerebrospinal fluid (CSF) biomarkers, and genetic data. Six peripheral inflammatory indices were derived: neutrophil-to-lymphocyte ratio (NLR), monocyte-to-lymphocyte ratio (MLR), platelet-to-lymphocyte ratio (PLR), systemic immune-inflammation index (SII), systemic inflammation response index (SIRI), and aggregate index of systemic inflammation (AISI). Spearman correlation, multiple linear regression, and generalized estimating equations were employed to examine associations. Unsupervised k-means clustering was performed to identify distinct inflammatory clusters, with differences assessed using ANCOVA analysis. NLR and SII were significantly elevated in PD, with NLR showing the strongest association. Peripheral inflammatory biomarkers showed distinct clinical correlations, with NLR demonstrating associations with both non-motor (cognitive decline, olfactory impairment, and depression, *p* < 0.001) and motor symptoms (*p* < 0.001). SII, and SIRI showed correlations with motor progression (*p* < 0.001), while SII additionally associated with sleepiness disorders (*p* < 0.001). Cluster analysis identified two distinct inflammatory clusters: a high-inflammation cluster demonstrating significantly worse cognitive function (*p* = 0.008), olfactory impairment (*p* < 0.001), and autonomic dysfunction (*p* < 0.001) at baseline, along with accelerated motor (*p* = 0.038) and cognitive decline (*p* < 0.001) during follow-up. This high-inflammation cluster also showed elevated CSF neurodegeneration markers including pTau, tTau, NfL, and GFAP (*p* < 0.05). Peripheral inflammatory biomarkers show robust associations with clinical features in PD, highlighting their potential as markers associated with disease features and progression, and suggesting a basis for inflammatory-based subgrouping.

**Supplementary Information:**

The online version contains supplementary material available at 10.1007/s10571-026-01708-8.

## Introduction

Parkinson’s disease (PD) is a complex neurodegenerative disorder affecting approximately 1% of the population aged over 65 years(Pringsheim et al. 2014). The global incidence of PD ranges from 10 to 18 per 100, 000 person-years, impacting over 6 million individuals worldwide(Tolosa et al. 2021), with projections suggesting this will rise to 25.2 million by 2050(Su et al. 2025). Traditionally, PD is characterized by bradykinesia, resting tremor, and rigidity - symptoms primarily caused by progressive degeneration of dopaminergic neurons in the nigrostriatal pathway(Kalia and Lang 2015). While the etiopathogenesis of PD remains incompletely understood, it is widely attributed to a complex interplay of aging, genetic susceptibility, and environmental factors. Recent evidence further implicates neuroinflammation as a key contributor to the occurrence and development of PD(Tansey et al. 2022).

Growing evidence indicates that central and peripheral inflammation, alongside other pathological processes, contribute to the initiation and progression of PD(Tansey et al. 2022; Williams et al. 2022). Within the central nervous system (CNS), misfolded α-synuclein can trigger immune activation, thereby exacerbating neuronal damage(Tansey and Romero-Ramos 2019). Both postmortem and in vivo studies demonstrate that neurodegeneration in the substantia nigra pars compacta (SNpc) occurs alongside microglial and astrocytic activation, accompanied by the release of inflammatory mediators and T-lymphocyte infiltration(Tansey and Romero-Ramos 2019; Folta et al. 2025). Furthermore, substantial evidence implicates peripheral inflammatory conditions in PD pathophysiology, with emerging findings suggesting bidirectional brain-periphery communication in this disease(Munoz-Delgado et al. 2023a; La Vitola et al. 2021). Compared to healthy controls (HCs), PD patients exhibit dysregulated cytokine profiles in both peripheral blood and cerebrospinal fluid (CSF) (Marogianni et al. 2020). Peripheral immune dysregulation in PD is also reflected in altered leukocyte counts and subpopulation distributions(Lin et al. 2025; Munoz-Delgado et al. 2021, 2023a). Notably, leukocyte imbalances, particularly involving lymphocytes, may impair protective immunity while promoting a pro-inflammatory milieu conducive to neurodegeneration(Tansey et al. 2022). Consequently, investigating peripheral inflammatory mechanisms is critical for elucidating PD pathogenesis.

Peripheral inflammatory markers derived from blood cell counts are widely recognized as non-invasive indicators of systemic inflammatory status, reflecting underlying peripheral immune activation. Peripheral inflammatory markers such as the neutrophil-to-lymphocyte ratio (NLR), platelet-to-lymphocyte ratio (PLR), and monocyte-to-lymphocyte ratio (MLR) have demonstrated prognostic value in neurological diseases including stroke, autoimmune encephalitis, Alzheimer’s disease, and other neurological diseases(Gong et al. 2021; Wang et al. 2023; Liu et al. 2022; Mohammadi et al. 2024; Zhou et al. 2022). In PD, studies have reported elevated NLR in patients compared to HCs(Munoz-Delgado et al. 2021; Xiao et al. 2025), with both NLR and PLR correlating significantly with motor and cognitive symptom progression in PD(Xiao et al. 2025; Li et al. 2024; Lin et al. 2025). More recently developed systemic inflammatory indices, such as systemic immune-inflammation index (SII), systemic inflammation response index (SIRI), aggregate index of systemic inflammation (AISI), and hemoglobin, albumin, lymphocyte, and platelet (HALP) score, have shown associations with diverse disease outcomes(Wang et al. 2023; Zinellu et al. 2023; Huang et al. 2024). However, longitudinal correlations between these indices and multidimensional PD progression remain insufficiently characterized. Additionally, while various methods exist for PD subtyping, no current classification approach incorporates peripheral inflammatory markers, despite their potential relevance in PD pathogenesis. This study therefore aims to comprehensively evaluate peripheral inflammatory biomarkers in PD, examining their associations with clinical features, disease progression, and their potential for identifying inflammatory-based subgroups.

## Method

### Participants

Study data were obtained from the Parkinson’s Progression Markers Initiative (PPMI) database, sponsored by the Michael J. Fox Foundation (MJFF)(Parkinson Progression Marker 2011). PPMI is a longitudinal, observational, multi-center natural history study launched in 2010. Its primary objective is to identify biomarkers of disease progression to accelerate therapeutic trials aimed at slowing PD-related disability. The study comprehensively assesses the progression of clinical features, imaging and biological markers, genetic data, and digital outcomes across all disease stages from prodromal to moderate PD. The PPMI clinical protocol enrolls approximately 4,000 participants (predominantly of Caucasian ancestry) across nearly 50 clinical sites in 12 countries throughout North America and Europe. The cohort includes individuals with prodromal PD, recently diagnosed PD patients, and healthy controls. Follow-up assessments are conducted every six months for the first five years and annually thereafter, with the majority of visits performed in person, supplemented by remote data collection. We downloaded comprehensive datasets in January 2025, including longitudinal clinical assessments, genetic data, and laboratory blood results. This study received approval from the PPMI Scientific Steering Committee. Analytical datasets can be accessed via the PPMI portal (https://ida.loni.usc.edu/login.jsp) following PPMI approval.

The inclusion criteria for PD were as follow: (1) diagnosis of PD without comorbid neurological disorders; (2) absence of prodromal PD or scans without evidence of dopaminergic deficit (SWEDDs), confirmed by abnormal dopamine transporter binding on ¹²³I-ioflupane SPECT imaging (DaTscan); (3) no regular use of non-steroidal anti-inflammatory drugs (NSAIDs) or corticosteroids; (4) completion of ≥ 5 annual study visits with ≤ 20% missing data (including baseline).

The inclusion criteria for HCs were as follows: (1) confirmed absence of PD and other neurological diseases; (2) no first-degree family history of PD; (3) no regular use of NSAIDs or corticosteroids; (4) completion of ≥ 5 annual study visits with ≤ 20% missing data (including baseline). A flow chart illustrating the patient selection process is presented in Supplementary Fig. 1. Following screening, the final cohort comprised 435 patients with PD and 207 HCs.

The diagnoses of PD and prodromal PD were based on the respective clinical criteria established by the Movement Disorder Society (MDS) (Postuma et al. 2015; Berg et al. 2015). Comorbidities, including conditions such as stroke, brain tumors, hydrocephalus, epilepsy, encephalitis, recurrent head trauma, and polyneuropathy, were identified through clinical evaluation. Medication history was recorded for all participants. No patient was receiving any PD-related medications at the baseline assessment.

### Clinical Evaluation

Demographic characteristics, including sex, age at enrollment, disease duration, comorbidities, and medication history, were obtained from all participants. Unified Parkinson’s Disease Rating Scale (UPDRS) part III (UPDRS-III) was used to assess motor symptoms. Non-motor symptoms were comprehensively assessed using standardized scales: the Montreal Cognitive Assessment (MoCA) for cognitive function, the Scales for Outcomes in Parkinson’s Disease-Autonomic (SCOPA-AUT) for autonomic dysfunction, and the Rapid Eye Movement Behavior Disorder Screening Questionnaire (RBDSQ) for REM sleep behavior disorder (RBD). Additional evaluations included the Epworth Sleepiness Scale (ESS) for daytime sleepiness, the Geriatric Depression Scale (GDS) for depression, the State-Trait Anxiety Inventory (STAI) for anxiety, the University of Pennsylvania Smell Identification Test (UPSIT) for olfactory function, and the Questionnaire for Impulsive-Compulsive Disorders (QUIP) for impulse control disorders. Demographic information was primarily self-reported by participants. All standardized clinical scales and assessments were administered and rated by trained clinicians. Participants underwent comprehensive baseline evaluations at enrollment, with standardized annual follow-up assessments of both motor and non-motor symptoms maintained for over five years.

### Acquisition of Biomarkers

#### Peripheral Inflammatory Markers

All biospecimen collection, processing, and laboratory analyses for this study were conducted according to the standardized protocols defined in the PPMI operations manual to ensure harmonization across all participating sites. Baseline samples were collected at PPMI clinical sites and processed centrally by the PPMI Biorepository Core. All subsequent laboratory analyses were performed uniformly by the PPMI Biorepository Core at Indiana University under rigorous quality control. Complete blood counts were determined by impedance spectroscopy, including: total leukocyte count (WBC), leukocyte subpopulations (neutrophils, lymphocytes, monocytes, eosinophils, and basophils), platelet count, hemoglobin, and albumin. Additionally, the following seven inflammatory indices were calculated from the hematological parameters: NLR ($\frac{\mathrm{n}\mathrm{e}\mathrm{u}\mathrm{t}\mathrm{r}\mathrm{o}\mathrm{p}\mathrm{h}\mathrm{i}\mathrm{l}\mathrm{s}}{\mathrm{l}\mathrm{y}\mathrm{m}\mathrm{p}\mathrm{h}\mathrm{o}\mathrm{c}\mathrm{y}\mathrm{t}\mathrm{e}\mathrm{s}}$), MLR ($\frac{\mathrm{m}\mathrm{o}\mathrm{n}\mathrm{o}\mathrm{c}\mathrm{y}\mathrm{t}\mathrm{e}\mathrm{s}}{\mathrm{l}\mathrm{y}\mathrm{m}\mathrm{p}\mathrm{h}\mathrm{o}\mathrm{c}\mathrm{y}\mathrm{t}\mathrm{e}\mathrm{s}}$ ), PLR ($\frac{\mathrm{p}\mathrm{l}\mathrm{a}\mathrm{t}\mathrm{e}\mathrm{l}\mathrm{e}\mathrm{t}\mathrm{s}}{\mathrm{l}\mathrm{y}\mathrm{m}\mathrm{p}\mathrm{h}\mathrm{o}\mathrm{c}\mathrm{y}\mathrm{t}\mathrm{e}\mathrm{s}}$ ), SII ($\frac{\mathrm{n}\mathrm{e}\mathrm{u}\mathrm{t}\mathrm{r}\mathrm{o}\mathrm{p}\mathrm{h}\mathrm{i}\mathrm{l}\mathrm{s}\times\mathrm{p}\mathrm{l}\mathrm{a}\mathrm{t}\mathrm{e}\mathrm{l}\mathrm{e}\mathrm{t}\mathrm{s}}{\mathrm{l}\mathrm{y}\mathrm{m}\mathrm{p}\mathrm{h}\mathrm{o}\mathrm{c}\mathrm{y}\mathrm{t}\mathrm{e}\mathrm{s}}$), SIRI ($\frac{\mathrm{n}\mathrm{e}\mathrm{u}\mathrm{t}\mathrm{r}\mathrm{o}\mathrm{p}\mathrm{h}\mathrm{i}\mathrm{l}\mathrm{s}\times\mathrm{m}\mathrm{o}\mathrm{n}\mathrm{o}\mathrm{c}\mathrm{y}\mathrm{t}\mathrm{e}\mathrm{s}}{\mathrm{l}\mathrm{y}\mathrm{m}\mathrm{p}\mathrm{h}\mathrm{o}\mathrm{c}\mathrm{y}\mathrm{t}\mathrm{e}\mathrm{s}}$ ), AISI ($\frac{\mathrm{n}\mathrm{e}\mathrm{u}\mathrm{t}\mathrm{r}\mathrm{o}\mathrm{p}\mathrm{h}\mathrm{i}\mathrm{l}\mathrm{s}\times\mathrm{p}\mathrm{l}\mathrm{a}\mathrm{t}\mathrm{e}\mathrm{l}\mathrm{e}\mathrm{t}\mathrm{s}\times\mathrm{m}\mathrm{o}\mathrm{n}\mathrm{o}\mathrm{c}\mathrm{y}\mathrm{t}\mathrm{e}\mathrm{s}}{\mathrm{l}\mathrm{y}\mathrm{m}\mathrm{p}\mathrm{h}\mathrm{o}\mathrm{c}\mathrm{y}\mathrm{t}\mathrm{e}\mathrm{s}}$), and HALP ($\frac{\mathrm{l}\mathrm{y}\mathrm{m}\mathrm{p}\mathrm{h}\mathrm{o}\mathrm{c}\mathrm{y}\mathrm{t}\mathrm{e}\mathrm{s}\times\mathrm{h}\mathrm{e}\mathrm{m}\mathrm{o}\mathrm{g}\mathrm{l}\mathrm{o}\mathrm{b}\mathrm{i}\mathrm{n}\times\mathrm{a}\mathrm{l}\mathrm{b}\mathrm{u}\mathrm{m}\mathrm{i}\mathrm{n}}{\mathrm{p}\mathrm{l}\mathrm{a}\mathrm{t}\mathrm{e}\mathrm{l}\mathrm{e}\mathrm{t}\mathrm{s}}$ ).

#### Cerebrospinal Fluid Biomarkers

CSF biomarkers previously investigated for PD diagnosis and progression were analyzed, including α-synuclein, phosphorylated tau (pTau), total tau (tTau), β-amyloid (Aβ), neurofilament light chain (NfL), and glial fibrillary acidic protein (GFAP). Data for these biomarkers, as assayed by ELISA, were acquired from the PPMI database.

### Genetic Information

Genetic data for PD-associated mutations, including LRRK2 (R1441G, G2019S), GBA (N409S, L483P), SNCA (A53T), and PRKN (R275W), were obtained from the PPMI database. After screening, a total of 104 LRRK2-PD, 67 GBA-PD, 19 SNCA-PD, and 8 PRKN-PD were enrolled in this study.

### Statistical Analysis

Statistical analyses were performed using IBM SPSS (version 26) and R software (version 4.3.0). Research procedures and statistical analyses were conducted under strict blinding protocols. Statisticians remained unaware of group identities until final model validation. This framework eliminated confirmation bias during both data collection and analytical interpretation. Multiple imputation was employed to handle missing data. All clinical assessment and laboratory data were standardized to Z-scores prior to analysis. The reference population for calculating the mean and standard deviation (SD) was chosen depending on the analytical context: the HC group was used for PD-HC comparisons, while the entire PD cohort served as the reference for within-PD analyses. For clinical interpretability, descriptive statistics and results in tables are reported using the original scale values. Homogeneity of variances was assessed using Levene’s test, while normality was evaluated via the Kolmogorov-Smirnov test (Supplementary Table 1). Quantitative data are presented as mean ± SD for normally distributed variables and median (interquartile range, IQR) for non-normally distributed variables. Between-group comparisons were performed using independent samples t-tests for normally distributed continuous data involving two groups, one-way ANOVA for three or more groups, and chi-square tests for categorical variables. Non-normally distributed continuous data were analyzed using the Mann-Whitney U test for two-group comparisons and the Kruskal-Wallis test for comparisons among three or more groups. Diagnostic performance of peripheral inflammatory markers in PD and HCs was evaluated through receiver operating characteristic (ROC) curve analysis. Spearman correlation analysis examined relationships between peripheral inflammatory markers, clinical features, and CSF biomarkers. The cross-sectional and longitudinal associations of peripheral inflammatory markers with clinical features and CSF biomarkers were addressed by multiple linear regression and generalized estimating equations (GEE), respectively. Disease duration, gender, age, and age at onset were chosen as covariates. To evaluate the robustness of our statistical models, we conducted sensitivity analyses for both regression approaches. For the linear regression models, we used Cook’s distance to identify influential observations and refitted the models after their exclusion. For the longitudinal GEE models, we assessed sensitivity to the working correlation structure by fitting models with exchangeable, autoregressive (AR1), and unstructured specifications.

To identify inflammatory clusters in PD, k-means clustering with Euclidean distance metrics was employed to identify data-driven inflammatory clusters, with optimal cluster number (k) determined via elbow plot analysis of total sum of squares (Supplementary Fig. 2). The k-means algorithm was run with 25 random initializations (nstart = 25) to ensure convergence to a stable solution. To assess the clustering quality, we calculated the silhouette coefficient. Cluster heterogeneity was subsequently evaluated using principal component analysis (PCA). The stability of the identified inflammatory clusters was assessed via bootstrap resampling (1000 iterations), quantified by calculating a consensus index that reflects the consistency of sample pair groupings across resampled datasets. Analysis of covariance (ANCOVA) was performed to compare clusters while controlling for disease duration, gender, age, and age at onset as covariates. All significance thresholds were adjusted using the Bonferroni correction for multiple comparisons.

## Results

### Comparison of Peripheral Inflammatory Markers Between PD and HCs

**Fig. 1:**
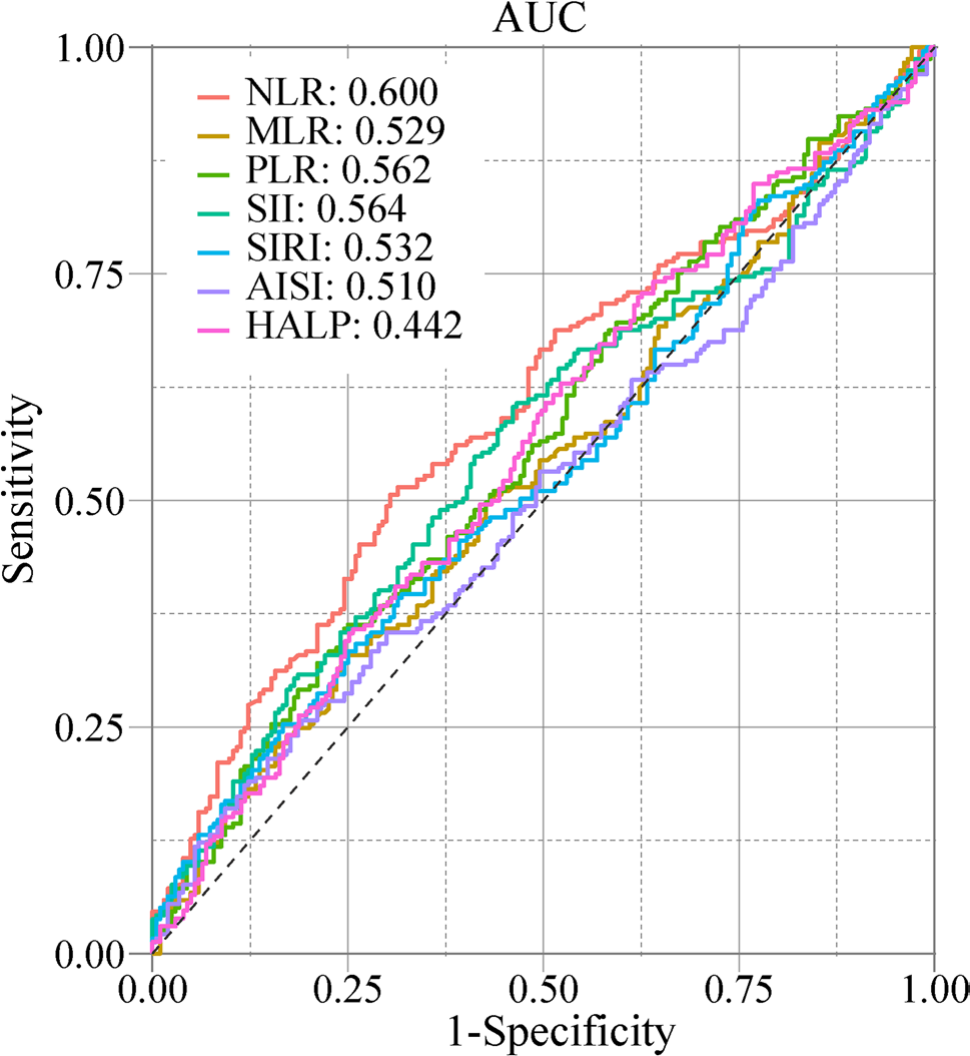
Discriminative ability of seven peripheral inflammatory biomarkers in distinguishing PD from HC using ROC curve analysis. Among all biomarkers evaluated, NLR demonstrated the highest discriminatory accuracy (AUC = 0.600). Abbreviations: NLR, neutrophil-to-lymphocyte ratio; PLR, platelet-to-lymphocyte ratio; MLR, monocyte-to-lymphocyte ratio; SII, systemic immune-inflammation index; SIRI, systemic inflammation response index; AISI, aggregate index of systemic inflammation; HALP, hemoglobin, albumin, lymphocyte, and platelet; ROC, receiver operating characteristic; AUC, area under curve

A total of 435 PD patients and 207 HCs were enrolled in this study. The primary analyses were conducted on 237 patients who were non-carriers of mutations in *LRRK2*, *GBA*, *SNCA*, or *PRKN* to minimize confounding from these known PD-related genes. Demographics and clinical characteristics of PD participants were presented in Supplementary Table 2. Peripheral inflammatory markers of NLR, MLR, PLR, SII, SIRI, AISI, and HALP were calculated for PD and HC (Table 1, Supplementary Fig. 3). Comparative analysis revealed significant elevations in NLR (*p* = 0.001) and SII (*p* = 0.023) among PD patients compared to HCs (Table 1, Supplementary Fig. 3), while MLR, PLR, SIRI, AISI, and HALP showed no significant differences. ROC analysis demonstrated that NLR showed the highest ability to discriminate between PD and HC groups among all inflammatory markers examined, albeit with a modest area under the curve (AUC) of 0.600 (*p* < 0.001, Fig. 1), with other inflammatory markers showing AUC values below 0.600 (Fig. 1).

**Table 1:** Comparison of peripheral inflammatory markers between PD and HC

Peripheral inflammatory markers	PD (*N* = 237)	HC (*N* = 207)	*P*
NLR	2.408(1.758–3.041)	2.064(1.623–2.680)	**0.001**
MLR	0.239(0.174–0.299)	0.227(0.176–0.279)	0.349
PLR	153.374(122.718-190.405)	146.222(113.333-177.895)	0.086
SII	560.000(375.616-750.534)	483.007(387.287-679.951)	**0.023**
SIRI	0.842(0.548–1.249)	0.815(0.535–1.128)	0.091
AISI	185.467(118.403-319.979)	182.159(130.788-286.747)	0.327
HALP	37.335(29.659–47.528)	40.228(32.366–52.259)	0.142

*NLR* Neutrophil-to-lymphocyte ratio, *MLR* Monocyte-to-lymphocyte ratio, *PLR* Platelet-to-lymphocyte ratio, *SII* Systemic immune-inflammation index, *SIRI* Systemic inflammation response index, *AISI* Aggregate index of systemic inflammation, *HALP* Hemoglobin, albumin, lymphocyte, and platelet, *PD* Parkinson’s disease, *HC* Healthy control. Mann-Whitney U test was used for analysis. Bold values indicate significant differences among groups (*p* < 0.05)

### Clinical Relevance of Peripheral Inflammatory Markers

**Fig. 2:**
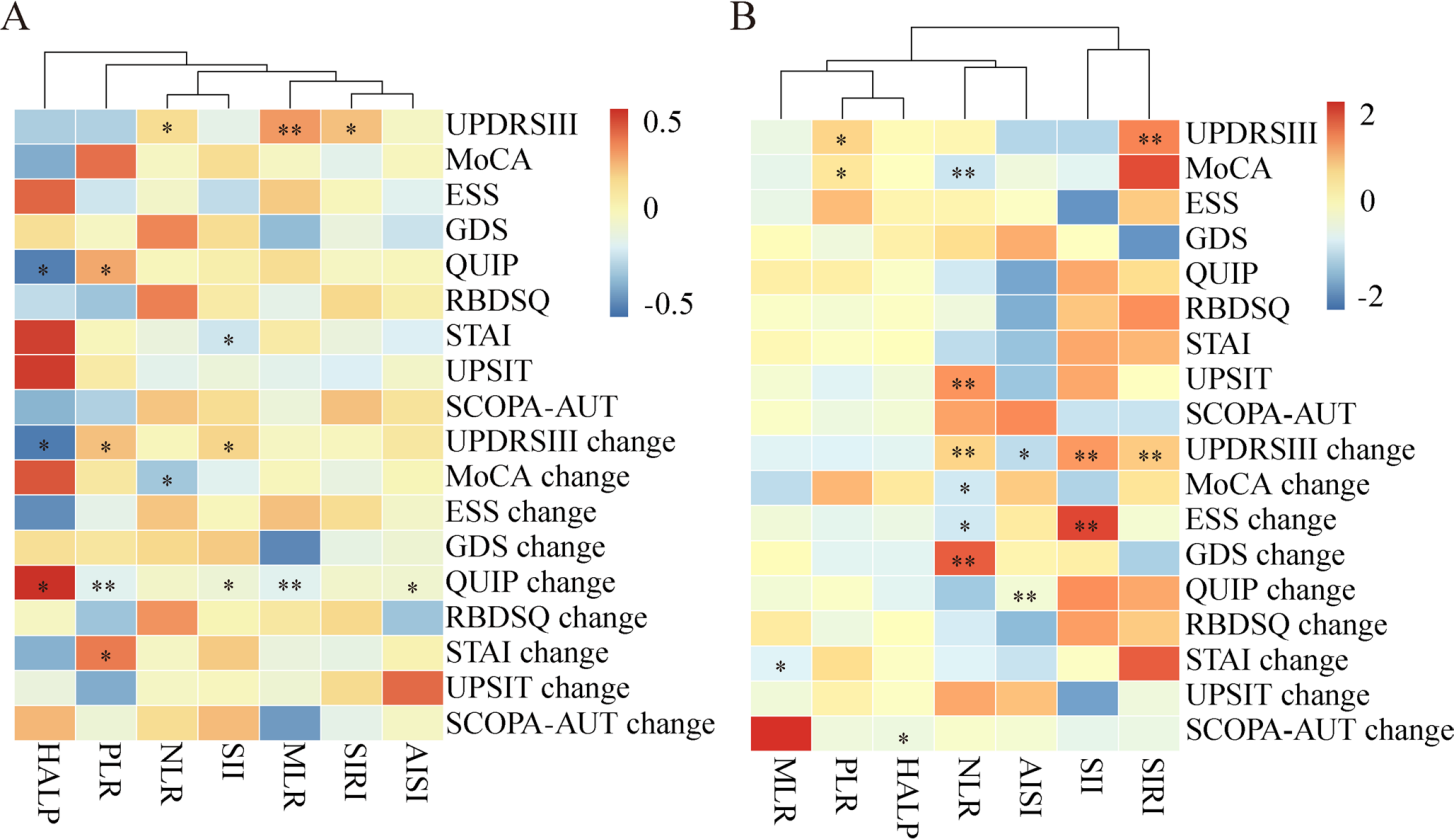
Correlations between peripheral inflammatory biomarkers and clinical features in PD. **A** Spearman correlation analysis evaluating relationships between inflammatory markers and clinical parameters. **B** Multiple linear regression and generalized estimating equation analysis evaluating relationships between inflammatory markers and clinical parameters. The darker the red, the stronger the positive correlation, while the darker the blue, the stronger the negative correlation. * *p* value passed general significance (*p* < 0.05); ** p passed Bonferroni correction (*p* < 0.007). Abbreviations: PD, Parkinson’s disease; UPDRS III, Unified Parkinson’s Disease Rating Scale part III; MoCA, Montreal Cognitive Assessment; ESS, Epworth Sleepiness Scale; GDS, Geriatric Depression Scale; QUIP, Questionnaire for Impulsive-Compulsive Disorders; RBDSQ, Rapid Eye Movement Behavior Disorder Screening Questionnaire; SCOPA-AUT, Scales for Outcomes in Parkinson’s disease - Autonomic; STAI, State-Trait Anxiety Inventory; UPSIT, University of Pennsylvania Smell Identification Test; NLR, neutrophil-to-lymphocyte ratio; PLR, platelet-to-lymphocyte ratio; MLR, monocyte-to-lymphocyte ratio; SII, systemic immune-inflammation index; SIRI, systemic inflammation response index; AISI, aggregate index of systemic inflammation; HALP, hemoglobin, albumin, lymphocyte, and platelet

In baseline evaluation, the correlation analysis demonstrated significant associations between inflammatory markers and clinical features (Supplementary Table 3, Fig. 2A): NLR, MLR and SIRI with motor symptoms; PLR and HALP with impulse control disorder; and SII with anxiety. After Bonferroni correction (α = 0.007), only MLR remained significantly associated with UPDRS-III (*p* < 0.007). Multiple linear regression analysis revealed additional possible correlations (*p* < 0.05): NLR with cognitive function and olfactory function; PLR with motor symptoms and cognitive function; and SIRI with motor symptoms (Table 2; Fig. 2B). After Bonferroni correction, NLR remained significantly associated with MoCA and UPSIT, and SIRI remained associated with UPDRS-III (all *p* < 0.001). Sensitivity analysis using Cook’s distance indicated no undue influence from individual observations, and the key associations remained unchanged upon their exclusion, supporting the robustness of our findings.

**Table 2:** Associations between peripheral inflammatory markers and clinical characteristics in PD

Clinical characteristics		NLR		MLR		PLR		SII		SIRI		AISI		HALP	
		Beta	*P*	Beta	*P*	Beta	*P*	Beta	*P*	Beta	*P*	Beta	*P*	Beta	*P*
Baseline Evaluation^a^	UPDRSIII	0.072	0.753	-0.167	0.275	0.316	**0.042**	-0.422	0.200	0.590	**< 0.001** ^*^	-0.419	0.195	0.053	0.515
	MoCA	-0.366	**< 0.001** ^*^	-0.245	0.116	0.145	**0.012**	-0.263	0.434	0.626	0.084	-0.171	0.604	-0.057	0.494
	ESS	0.087	0.715	-0.102	0.518	0.278	0.122	-0.429	0.204	0.250	0.494	0.014	0.967	0.091	0.277
	GDS	0.124	0.604	0.025	0.876	-0.042	0.817	0.009	0.98	-0.265	0.471	0.191	0.568	0.065	0.440
	QUIP	-0.196	0.407	0.091	0.559	0.087	0.628	0.286	0.396	0.175	0.630	-0.391	0.237	-0.026	0.755
	RBDSQ	-0.102	0.668	-0.038	0.807	-0.066	0.712	0.227	0.502	0.338	0.356	-0.400	0.230	-0.020	0.810
	STAI	-0.336	0.158	0.032	0.838	-0.036	0.842	0.352	0.300	0.315	0.391	-0.438	0.189	-0.009	0.913
	UPSIT	0.472	**< 0.001** ^*^	0.048	0.759	-0.059	0.743	0.424	0.211	0.112	0.759	-0.230	0.489	0.026	0.760
	SCOPA-AUT	0.324	0.173	-0.043	0.786	-0.123	0.495	-0.270	0.425	-0.276	0.450	0.380	0.254	-0.090	0.282
Longitudinal Evaluation^b^	UPDRSIII	0.041	**< 0.001** ^*^	0.014	0.260	0.013	0.087	0.051	**< 0.001** ^*^	0.043	**< 0.001** ^*^	0.008	**0.034**	0.015	0.425
	MoCA	-0.051	**0.018**	-0.061	0.179	0.071	0.098	-0.070	0.552	0.040	0.765	0.058	0.647	0.033	0.078
	ESS	-0.085	**0.047**	0.044	0.219	-0.026	0.750	0.609	**< 0.001** ^*^	0.065	0.109	0.236	0.830	0.009	0.112
	GDS	0.041	**< 0.001** ^*^	0.000	0.996	-0.016	0.620	0.007	0.956	-0.030	0.774	0.004	0.974	-0.016	0.319
	QUIP	-0.078	0.245	-0.013	0.687	-0.002	0.953	0.098	0.296	0.083	0.313	-0.014	**< 0.001** ^*^	-0.035	0.270
	RBDSQ	-0.043	0.71	0.031	0.524	-0.021	0.070	0.080	0.617	0.056	0.679	-0.080	0.635	0.006	0.634
	STAI	-0.077	0.446	-0.078	**0.039**	0.069	0.054	-0.008	0.955	0.182	0.095	-0.098	0.437	-0.012	0.289
	UPSIT	0.258	0.325	-0.067	0.620	0.069	0.603	-0.364	0.347	-0.078	0.788	0.214	0.493	-0.007	0.831
	SCOPA-AUT	0.107	0.327	1.329	0.007	-0.005	0.910	-0.110	0.453	-0.058	0.657	0.047	0.747	-0.025	**0.044**

*PD* Parkinson’s disease, *UPDRS III* Unified Parkinson’s Disease Rating Scale part III, *MoCA* Montreal Cognitive Assessment, *ESS* Epworth Sleepiness Scale, *GDS* Geriatric Depression Scale, *QUIP* Questionnaire for Impulsive-Compulsive Disorders, *RBDSQ* Rapid Eye Movement Behavior Disorder Screening Questionnaire, *SCOPA-AUT* Scales for Outcomes in Parkinson’s disease - Autonomic, *STAI* State-Trait Anxiety Inventory, *UPSIT* University of Pennsylvania Smell Identification Test, *NLR* Neutrophil-to-lymphocyte ratio, *PLR* Platelet-to-lymphocyte ratio, *MLR* Monocyte-to-lymphocyte ratio, *SII* Systemic immune-inflammation index, *SIRI* Systemic inflammation response index, *AISI* Aggregate index of systemic inflammation, *HALP* Hemoglobin, albumin, lymphocyte, and platelet. The reported β coefficients represent the mean difference in the outcome per unit change in the predictor. Bold values indicate significant differences among groups (*p* < 0.05). ^a^ Multiple linear regression was used for analysis. ^b^ Generalized estimating equation was used for analysis. * *p* < 0.007 was taken as cutoff value for significance after multiple correction

Longitudinal analysis demonstrated significant associations between inflammatory markers and disease progression measures: PLR, SII and HALP correlated with UPDRS-III changes; NLR with MoCA changes; MLR, PLR, SII, AISI and HALP with QUIP changes; and PLR with STAI changes (Supplementary Table 3, Fig. 2A). Following multiple comparison correction (α = 0.007), only MLR (*p* = 0.006) and PLR (*p* = 0.006) maintained significant associations with QUIP changes. GEE analysis revealed additional possible correlations (*p* < 0.05): NLR with motor, cognitive, sleepiness, and depression measures; MLR with anxiety; SII with motor and sleepiness decline; AISI with motor and impulse control; and HALP with autonomic function. After multiplicity adjustment, NLR remained associated with motor decline and depression, SII with motor and sleepiness decline, and AISI with impulse control (all *p* < 0.001, Table 2; Fig. 2B). Sensitivity analysis confirmed that the associations of inflammatory markers with clinical progression were consistent in direction, magnitude, and statistical significance across all correlation structures, supporting the robustness of our primary findings.

### Associations Between Peripheral Inflammatory Markers and Cerebrospinal Fluid Biomarkers

CSF analysis was performed on 231 out of the 237 PD participants, as 6 individuals did not undergo the lumbar puncture procedure. Initial correlation analysis revealed no significant associations between peripheral inflammatory markers and CSF biomarkers (Supplementary Table 4, Supplementary Fig. 4A). Subsequent multiple linear regression analysis identified associations (Supplementary Table 5, Supplementary Fig. 4B), with elevated NLR corresponding to higher Nfl levels (*p* = 0.020) and increased SIRI associated with lower α-syn levels (*p* < 0.001). Following Bonferroni correction, SIRI remained significant associations with α-syn.

### Genetic Variation in Peripheral Inflammatory Markers

Our cohort comprised 104 LRRK2 (G2019S/R1441C), 67 GBA (L444P/N370S), 19 SNCA (A53T), and 8 PRKN (R275W) mutation carriers with PD. Comparative analysis revealed no significant differences in all 7 peripheral inflammatory markers between mutation carriers and non-carriers after Bonferroni correction (α = 0.007, Supplementary Table 6, Supplementary Fig. 5). Notably, in exploratory subgroup analyses, *LRRK2* carriers displayed distinct peripheral inflammatory patterns, showing significantly lower PLR (*p* = 0.005) and higher HALP (*p* = 0.012) compared to non-carriers (Supplementary Table 6, Supplementary Fig. 5).

### Inflammatory Clustering and Clinical Associations of PD

**Fig. 3:**
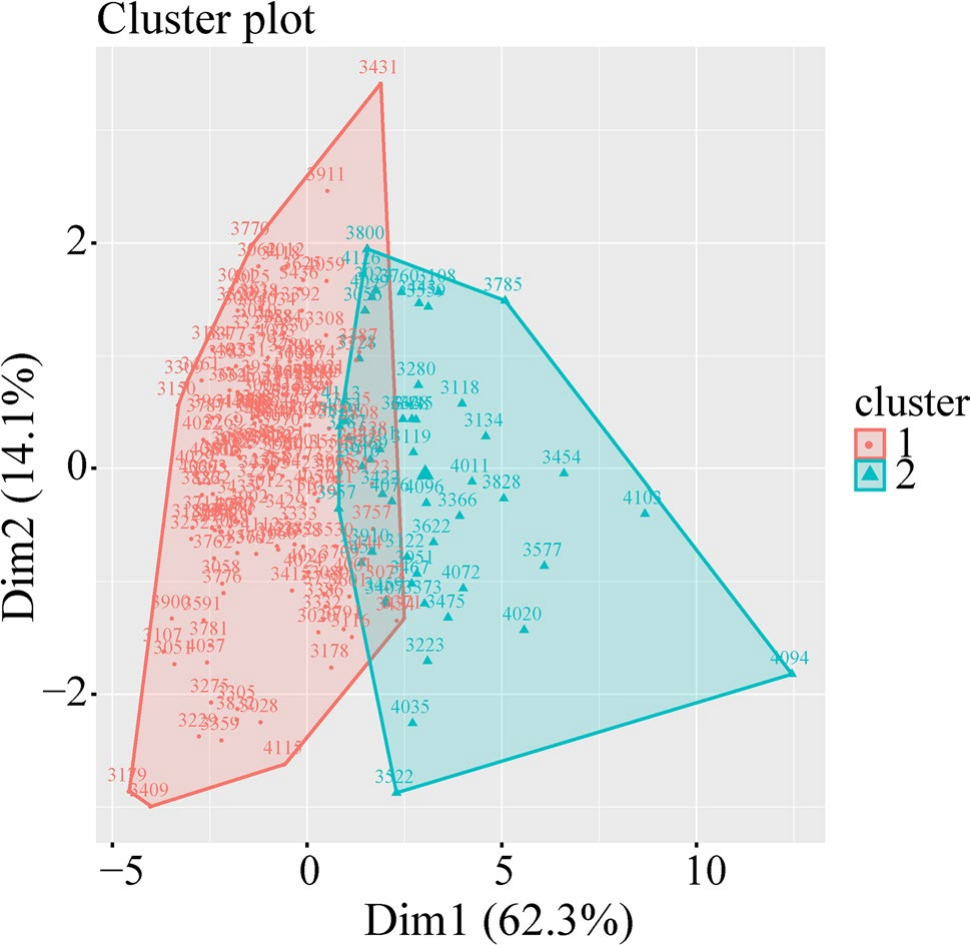
Distribution of cluster memberships through PCA analysis. Red represents cluster 1, green represents cluster 2. Significant differences in principal components among individuals of each cluster

K-means clustering stratified PD patients into two distinct inflammatory clusters: low-inflammation (cluster 1, *N* = 133) and high-inflammation (cluster 2, *N* = 104) subgroups. The overall average silhouette width was 0.512, indicating a fair cluster structure. The bootstrap analysis indicated robust cluster stability, yielding consensus indices of 0.773 and 0.985 for Cluster 1 and Cluster 2, respectively. Cluster 2 demonstrated significantly elevated peripheral inflammatory markers versus cluster 1 (*p* < 0.001, Supplementary Table 7), with clear separation confirmed PCA analysis (Fig. 3). At baseline, Cluster 2 exhibited worse cognitive function (*p* = 0.008), olfactory impairment (*p* < 0.001), and autonomic dysfunction (*p* < 0.001) compared to cluster 1 (Table 3). Longitudinally, cluster 2 showed accelerated motor progression (*p* = 0.038) and cognitive decline (*p* < 0.001) compared to cluster 1 (Table 3). In addition, patients in the high-inflammation cluster (Cluster 2) demonstrated significantly elevated levels of pTau, tTau, NfL, and GFAP compared to cluster 1 (Supplementary Table 8). No significant differences were observed in Aβ42 or α-synuclein levels between clusters.

**Table 3:** Clinical characteristics between low-inflammation (Cluster 1) and high-inflammation (Cluster 2) patient groups

Clinical characteristics		Cluster 1 (*N* = 133)	Cluster 2 (*N* = 104)	*P*
Baseline Evaluation	UPDRSIII	18.750 ± 7.930	20.720 ± 8.739	0.078
	MoCA	28(27–30)	27(25.5–29)	**0.008**
	ESS	5.82 ± 3.293	5.61 ± 3.353	0.636
	GDS	5.300 ± 1.574	5.190 ± 1.410	0.559
	QUIP	4(4–4)	4(4–4)	0.405
	RBDSQ	4.070 ± 2.685	4.360 ± 2.734	0.421
	STAI	92.350 ± 8.307	93.210 ± 7.819	0.422
	UPSIT	25.130 ± 7.662	19.660 ± 7.034	**< 0.001**
	SCOPA-AUT	8(5–15)	13(9-21.5)	**< 0.001**
Longitudinal Evaluation	UPDRSIII change	0.956 ± 2.340	1.636 ± 2.546	**0.038**
	MoCA change	0(-0.2-0.4)	-0.2(-0.6-0.2)	**< 0.001**
	ESS change	0.367 ± 0.898	0.467 ± 0.802	0.379
	GDS change	0.027 ± 0.333	0.061 ± 0.365	0.477
	QUIP change	-0.8(-0.8–0.4)	-0.8(-0.8–0.2)	0.779
	RBDSQ change	0(-0.2-0.4)	0.2(-0.2-0.6)	0.318
	STAI change	-0.008 ± 1.757	-0.344 ± 1.858	0.173
	UPSIT change	-1(-2.4–0.4)	-0.4(-0.9-0.2)	**0.018**
	SCOPA-AUT change	0.858 ± 1.769	1.366 ± 2.259	0.070

*PD* Parkinson’s disease, *UPDRS III* Unified Parkinson’s Disease Rating Scale part III, *MoCA* Montreal Cognitive Assessment, *ESS* Epworth Sleepiness Scale, *GDS* Geriatric Depression Scale, *QUIP* Questionnaire for Impulsive-Compulsive Disorders, *RBDSQ* Rapid Eye Movement Behavior Disorder Screening Questionnaire, *SCOPA-AUT* Scales for Outcomes in Parkinson’s disease - Autonomic, *STAI* State-Trait Anxiety Inventory, *UPSIT* University of Pennsylvania Smell Identification Test, *NLR* Neutrophil-to-lymphocyte ratio, *PLR* Platelet-to-lymphocyte ratio, *MLR* Monocyte-to-lymphocyte ratio *SII* Systemic immune-inflammation index, *SIRI* Systemic inflammation response index, *AISI* Aggregate index of systemic inflammation, *HALP* Hemoglobin, albumin, lymphocyte, and platelet. ANCOVA and Mann-Whitney U test were used for analysis. Bold values indicate significant differences among groups (*p* < 0.05)

## Discussion

This study demonstrates significant associations between peripheral inflammatory markers and clinical features in PD. These findings suggest that inflammatory markers may serve as indicators of disease severity and progression, and provide a basis for further investigating inflammatory-based subgroups. We found that specific markers like NLR and SII could distinguish PD patients from HCs, while comprehensive inflammatory profiling revealed distinct clinical trajectories. Patients with high-inflammatory profiles may exhibit more severe motor and non-motor symptoms, faster disease progression, and elevated neurodegeneration-associated CSF biomarkers. These findings suggest that peripheral inflammation monitoring may help characterize disease heterogeneity and inform future research on targeted approaches in PD management.

Growing evidence underscores the critical role of peripheral inflammation in PD pathophysiology (Tansey et al. 2022; Williams et al. 2022; Munoz-Delgado et al. 2023a; La Vitola et al. 2021). Beyond cytokine involvement, emerging research highlights significant alterations in peripheral immune cells, including quantitative and qualitative changes in leukocyte subpopulations(Lin et al. 2025; Munoz-Delgado et al. 2021, 2023a; DeLong et al. 2022; Yang et al. 2020). Additionally, leukocyte-derived inflammatory markers have demonstrated robust associations with risk, disease severity, progression rates, and clinical subtypes in neurological disorders including PD(Gong et al. 2021; Wang et al. 2023; Liu et al. 2022; Mohammadi et al. 2024; Zhou et al. 2022). These findings collectively position peripheral inflammation as a process closely associated with PD pathogenesis. Further investigation of PD-inflammatory marker relationships remains essential for elucidating the role of inflammation in disease mechanisms and potential therapeutic targeting.

Our study confirmed that NLR was significantly elevated in PD patients compared to HCs and showed the strongest association with PD status among the inflammatory markers assessed. Clinically, elevated NLR correlated with cognitive decline and impaired olfaction at baseline. Longitudinal analysis further showed NLR’s association with worsening motor symptoms and depression progression. These findings suggest that NLR may appear to be one of the most promising inflammatory biomarkers. However, its modest discriminative ability indicates that inflammatory markers alone are insufficient for reliable disease identification. Future models combining NLR with other clinical and laboratory parameters may improve differential diagnostic performance.

NLR has emerged as the most extensively investigated peripheral inflammatory biomarker with established utility across various neurological disorders (Gong et al. 2021; Wang et al. 2023; Liu et al. 2022; Mohammadi et al. 2024; Zhou et al. 2022). In PD pathogenesis, NLR reflects the immunological disturbances of lymphocytes and neutrophils. Reductions in circulating lymphocytes in PD patients have been reported in multiple studies(Munoz-Delgado et al. 2021; Jiang et al. 2017; Garfias et al. 2022). These decreased levels of lymphocytes results from selective depletion of immunoregulatory cell populations, including CD4 + T-helper cells, CD19 + B-cells, and regulatory T cells (Tregs) (Jensen et al. 2021; Cen et al. 2017). The degree of lymphocyte loss correlates with nigrostriatal dopaminergic degeneration observed in neuroimaging studies(Lin et al. 2014; Munoz-Delgado et al. 2023a), suggesting a potential link between peripheral immune alterations and central neurodegeneration. Evidence further supports the neuroprotective role of lymphocytes, as experimental models demonstrate that lymphocyte activation enhances microglial phagocytic function and promotes α-synuclein clearance in the substantia nigra(Bido et al. 2024). Emerging evidence indicates that elevated neutrophil represents an early pathogenic event in PD, detectable years before clinical diagnosis(Lauritsen and Romero-Ramos 2023; Craig et al. 2021). Additionally, neutrophil level is associated with glymphatic system dysfunction and motor symptom(Lin et al. 2025; Kim et al. 2023). However, the precise mechanisms through which neutrophils contribute to PD progression remain incompletely understood. In summary, NLR provides unique clinical value by integrating two complementary immunological parameters: lymphocyte, reflecting impaired immunoregulatory capacity, and neutrophil, indicating systemic oxidative stress and inflammatory cytokine release. As a composite biomarker, NLR overcomes the limitations of individual leukocyte subtype measurements by capturing the dynamic balance between these opposing immune pathways. This integrative approach has been validated in PD, with studies and meta-analyses demonstrating consistent NLR elevation in patients compared to HCs(Munoz-Delgado et al. 2021; Hosseini et al. 2023). Furthermore, NLR correlates with motor subtype, clinical severity, and disease progression in PD(Li et al. 2024; Grillo et al. 2023; Xiao et al. 2025), which aligns with and is reinforced by our current study. The ability to simultaneously assess both the loss of regulatory function and the rise of pro-inflammatory activity makes it a promising tool for evaluating PD-related immune dysfunction and its clinical consequences.

Our findings also demonstrate a significant association between elevated peripheral NLR and increased NfL levels. As a neuron-specific component of the axonal cytoskeleton, NfL is released into extracellular fluid following axonal injury or neuronal death and has been recognized as a promising biomarker in PD (Olsson et al. 2019). Although the exact interplay between NfL and neuroinflammation remains unclear, emerging evidence indicates that NfL may not only result from inflammatory processes and blood-brain barrier disruption(Liu et al. 2024), but could also actively exacerbate neuroinflammation by activating microglia(Kahn et al. 2025). Together, these insights suggest that elevated NLR may reflect not only systemic inflammation but also indirect evidence of associated neuronal damage. Further studies are needed to elucidate the precise mechanisms linking peripheral inflammation, NfL, and neuroinflammatory pathways in PD.

Monocyte counts are elevated in both prodromal and clinical PD compared to HCs, yet these cells exhibit decreased functional vitality(Grozdanov et al. 2014; Lauritsen and Romero-Ramos 2023; Nissen et al. 2019). Concurrently, PD patients show reduced platelet counts with characteristic structural changes, including increased swelling volume, twisted morphology, and decreased membrane invagination(Beura et al. 2022). These findings support the potential utility of MLR and PLR as PD biomarkers. Specifically, we observed that MLR correlates with baseline motor symptoms and is associated with impulse control disorders during longitudinal follow-up, consistent with previous reports(Li et al. 2024). While some studies report PLR differences between PD and HC(Wang et al. 2024), our results showed limited PLR correlation with PD features, likely because both platelets and lymphocytes decrease in parallel, creating uncertainty in ratio interpretation.

Our study investigated several emerging peripheral inflammatory indices in PD, including SII, SIRI, AISI, and HALP. These biomarkers extend beyond traditional NLR by incorporating additional hematological parameters: SII integrates platelet counts, SIRI includes monocytes, and AISI combines both platelets and monocytes. We found elevated SII levels in PD patients compared to HCs, confirming previous reports(Zhao et al. 2025). Notably, SII, SIRI, and AISI all demonstrated associations with motor progression, highlighting their potential as markers associated with motor function decline. Furthermore, SIRI was specifically linked to both baseline motor symptoms and CSF α-syn levels. Given that α-syn is a core pathological protein in PD and its CSF level is a highly sensitive and specific biomarker reflecting clinical and pathological disease burden(Mollenhauer et al. 2017), the association with SIRI suggests that it may indirectly reflect the underlying pathological burden of PD. This connection warrants further investigation.

HALP further considered hemoglobin and albumin. Hemoglobin regulates iron and mitochondrial homeostasis in dopaminergic neurons, and higher levels of hemoglobin may increase PD risk by promoting iron accumulation(Freed and Chakrabarti 2016). Albumin exerts neuroprotection through antioxidant effects and glial modulation, with higher levels correlating with better motor and cognitive outcomes in PD(Prajapati et al. 2011; Sun et al. 2022). While higher HALP levels predict reduced mortality risk in PD(Jia et al. 2024), our study found no significant clinical associations. The differential performance of these indices underscores the complex interplay between systemic inflammation and neurodegenerative processes in PD.

Given the established role of genetic factors in PD pathogenesis, we investigated peripheral inflammatory biomarkers across different genetic subtypes. Our analysis revealed that patients with LRRK2 mutations exhibit distinct inflammatory profiles characterized by significantly lower PLR and higher HALP compared to non-carriers. These findings align with existing literature demonstrating differential peripheral immune responses between sporadic and genetic PD forms, particularly in LRRK2-PD(Munoz-Delgado et al. 2023b). Previous reports of elevated HALP in familial PD and increased levels of IL-1β and other pro-inflammatory cytokines in LRRK2-PD further support our observations(Zhou et al. 2025; Dzamko et al. 2016). Our study further supports that peripheral inflammatory pattern differs in PD, particular LRRK2-PD, and PLR and HALP may show distinct patterns in genetic subtyping. Collectively, these results underscore that peripheral inflammatory patterns differ substantially in PD, especially in LRRK2-PD, and suggest that PLR and HALP may serve as potential indicators for detecting specific genetic mutations.

Our study introduces a novel inflammatory stratification method for PD, classifying patients into high-inflammation and low-inflammation subgroups based on peripheral inflammatory biomarkers. This approach addresses limitations of conventional subtyping methods that relied on arbitrary age cutoffs (Wickremaratchi et al. 2011; Qian and Huang 2019) or unstable motor symptom patterns(Simuni et al. 2016). While recent data-driven methods have incorporated diverse biomarkers(Fereshtehnejad et al. 2017; Mestre et al. 2021), our work represents the first inflammatory-based classification. The identified subgroups exhibited significant differences in both clinical severity and progression rates for motor and non-motor symptoms, demonstrating a significant link between peripheral inflammation and clinical heterogeneity in PD. These findings not only provide a more stable and biologically-grounded classification system but also highlight the potential for personalized therapeutic strategies targeting specific inflammatory pathways. Future subtyping efforts should prioritize integration of inflammatory markers to better capture the complex interplay between immune dysfunction and neurodegeneration in PD.

This study has several limitations that warrant consideration. First, the observational nature of our analyses limits definitive causal inference regarding the role of peripheral inflammation in PD, and reverse causality cannot be ruled out. Second, peripheral inflammatory markers are indirect proxies for central neuroinflammation and may be influenced by systemic conditions in PD. Third, we cannot fully exclude potential residual confounding by unmeasured factors (detailed comorbidities or medications), and the exclusion of participants due to missing data or procedures may introduce selection bias, both of which could influence our results. Stronger biological rationale and more rigorous confounding control would be required to support our mechanistic or clinical claims. Fourth, while statistically significant, the discriminative value of certain peripheral inflammatory markers remains modest. This highlights the need for future research to develop composite models that augment inflammatory markers with additional clinical, imaging, or genetic data to achieve meaningful diagnostic or prognostic utility. Furthermore, our cohort primarily consisted of early-stage patients, which may affect generalizability to advanced stages. Finally, we define PD-associated inflammatory profiles. The specificity and generalizability of these profiles must be validated in larger independent cohorts and verified through comparison with other neurodegenerative conditions.

## Conclusion

Our study demonstrates significant associations between peripheral inflammatory markers and clinical features in PD. These findings suggest that peripheral inflammatory profiles are closely linked to PD pathogenesis and may serve as indicators of disease severity and progression. The identification of distinct inflammatory subgroups highlights the heterogeneity of immune dysregulation in PD and provides a basis for future research into personalized approaches. These results underscore the relevance of peripheral inflammation in PD and support further investigation into its underlying mechanisms and potential therapeutic implications.

## Supplementary Information

Below is the link to the electronic supplementary material.

Supplementary Material 1

Supplementary Material 2—Study participant inclusion and exclusion flowchart. Abbreviations: PPMI, Parkinson's Progression Markers Initiative; SWEDDs, scans without evidence of dopaminergic deficit; NSAIDs, non-steroidal anti-inflammatory drugs

Supplementary Material 3—Selection of optimal cluster number through elbow plots. The optimal cluster number (k=2) was determined via elbow plot analysis of total sum of squares

Supplementary Material 4—Comparison of peripheral inflammatory biomarker levels between PD and HC. **A** NLR; **B** MLR; **C** PLR; **D** SII; **E** SIRI; **F** AISI; **G** HALP. Abbreviations: NLR, neutrophil-to-lymphocyte ratio; PLR, platelet-to-lymphocyte ratio; MLR, monocyte-to-lymphocyte ratio; SII, systemic immune-inflammation index; SIRI, systemic inflammation response index; AISI, aggregate index of systemic inflammation; HALP, hemoglobin, albumin, lymphocyte, and platelet; ns, not significant. * p<0.05; *** p<0.001

Supplementary Material 5—Associations between peripheral inflammatory biomarkers and CSF biomarkers in PD. **A** Spearman correlation analysis of peripheral inflammatory markers with CSF biomarker levels. **B** Multiple linear regression analysis of peripheral inflammatory markers with CSF biomarker levels. The darker the red, the stronger the positive correlation, while the darker the blue, the stronger the negative correlation. * p value passed general significance (p<0.05); ** p passed Bonferroni correction (p<0.007). Abbreviations: NLR, neutrophil-to-lymphocyte ratio; MLR, monocyte-to-lymphocyte ratio; PLR, platelet-to-lymphocyte ratio; SII, systemic immune-inflammation index; SIRI, systemic inflammation response index; AISI, aggregate index of systemic inflammation; HALP, hemoglobin, albumin, lymphocyte, and platelet; α-syn, α-synuclein; pTau, phosphorylated tau; tTau ,total tau; Aβ, β-amyloid; NfL, neurofilament light chain; GFAP, glial fibrillary acidic protein

Supplementary Material 6—Comparison of peripheral inflammatory biomarker levels in PD mutation carriers. **A** NLR; **B** MLR; **C** PLR; **D** SII; **E** SIRI; **F** AISI; **G** HALP. Abbreviations: NLR, neutrophil-to-lymphocyte ratio; MLR, monocyte-to-lymphocyte ratio; PLR, platelet-to-lymphocyte ratio; SII, systemic immune-inflammation index; SIRI, systemic inflammation response index; AISI, aggregate index of systemic inflammation; HALP, hemoglobin, albumin, lymphocyte, and platelet. ns, not significant. * p<0.05

## Data Availability

The primary data supporting this study are available through the PPMI under controlled access (www.ppmi-info.org). Analytical datasets can be accessed via the PPMI portal (https://ida.loni.usc.edu/home/projectPage.jsp? project=PPMI) following PPMI approval.
