# Supplementary material for: Peripheral Inflammatory Biomarkers in Parkinson’s Disease: Clinical Correlations and Stratification
Source: Cell Mol Neurobiol. 2026 Mar 8;46:73. doi: 10.1007/s10571-026-01708-8 (PMC13009453; doi:10.1007/s10571-026-01708-8)
Supplement: Supplementary file 1 — Supplementary Material 1 [file 10571_2026_1708_MOESM1_ESM.docx]

**Supplementary Table 1**. **Normality and variance homogeneity assessments**

| Variables | Variance homogeneity test | Normality test |
| --- | --- | --- |
| NLR | 0.020 | <0.001 |
| MLR | 0.580 | <0.001 |
| PLR | 0.663 | <0.001 |
| SII | 0.066 | <0.001 |
| SIRI | 0.012 | <0.001 |
| AISI | 0.040 | <0.001 |
| HALP | 0.276 | <0.001 |
| UPDRSIII | 0.359 | 0.462 |
| MoCA | 0.947 | 0.007 |
| ESS | 0.779 | 0.090 |
| GDS | 0.220 | 0.112 |
| QUIP | 0.992 | <0.001 |
| RBDSQ | 0.452 | 0.129 |
| STAI | 0.243 | 0.396 |
| UPSIT | 0.357 | 0.130 |
| SCOPA-AUT | 0.013 | 0.027 |
| UPDRSIII change | 0.296 | 0.262 |
| MoCA change | <0.001 | 0.677 |
| ESS change | 0.224 | 0.560 |
| GDS change | 0.115 | 0.092 |
| QUIP change | 0.289 | <0.001 |
| RBDSQ change | 0.998 | 0.001 |
| STAI change | 0.851 | 0.351 |
| UPSIT change | 0.031 | <0.001 |
| SCOPA-AUT change | 0.116 | 0.782 |
| Aβ42 | 0.540 | 0.698 |
| pTau | 0.131 | 0.178 |
| tTau | 0.016 | 0.603 |
| α-synuclein | 0.046 | 0.015 |
| NfL | 0.019 | 0.003 |
| GFAP | 0.047 | 0.346 |

Abbreviations: NLR, neutrophil-to-lymphocyte ratio; PLR, platelet-to-lymphocyte ratio; MLR, monocyte-to-lymphocyte ratio; SII, systemic immune-inflammation index; SIRI, systemic inflammation response index; AISI, aggregate index of systemic inflammation; HALP, hemoglobin, albumin, lymphocyte, and platelet; UPDRS III, Unified Parkinson’s Disease Rating Scale part III; MoCA, Montreal Cognitive Assessment; ESS, Epworth Sleepiness Scale; GDS, Geriatric Depression Scale; QUIP, Questionnaire for Impulsive-Compulsive Disorders; RBDSQ, Rapid Eye Movement Behavior Disorder Screening Questionnaire; SCOPA-AUT, Scales for Outcomes in Parkinson’s disease - Autonomic; STAI, State-Trait Anxiety Inventory; UPSIT, University of Pennsylvania Smell Identification Test.  Levene's test assessed variance homogeneity and Kolmogorov-Smirnov test evaluated normality.

**Supplementary Table 2**. **Demographic and clinical characteristics of PD at baseline**

| **Clinical characteristics** | **PD** |
| --- | --- |
| Gender(male/female) | 151/86 |
| Age | 60.82±9.27 |
| Disease duration (month) | 16.50(10-30) |
| UPDRS III | 20.00±8.61 |
| MoCA | 28(26-29) |
| ESS | 5.69±3.32 |
| GDS | 5.22±1.48 |
| QUIP | 4(4-4) |
| RBDSQ | 4.25±2.72 |
| STAI | 92.89±7.96 |
| UPSIT | 21.93±7.77 |
| SCOPA-AUT | 11(7-19) |

Abbreviations: PD, Parkinson’s disease; UPDRS III, Unified Parkinson’s Disease Rating Scale part III; MoCA, Montreal Cognitive Assessment; ESS, Epworth Sleepiness Scale; GDS, Geriatric Depression Scale; QUIP, Questionnaire for Impulsive-Compulsive Disorders; RBDSQ, Rapid Eye Movement Behavior Disorder Screening Questionnaire; SCOPA-AUT, Scales for Outcomes in Parkinson’s disease - Autonomic; STAI, State-Trait Anxiety Inventory; UPSIT, University of Pennsylvania Smell Identification Test.

**Supplementary Table 3**. **Associations between peripheral inflammatory markers and clinical characteristics in PD**

| **Clinical characteristics** | | **NLR** |  | **MLR** |  | **PLR** |  | **SII** |  | **SIRI** |  | **AISI** |  | **HALP** |  |
| --- | --- | --- | --- | --- | --- | --- | --- | --- | --- | --- | --- | --- | --- | --- | --- |
|  |  | R | *P* | R | *P* | R | *P* | R | *P* | R | *P* | R | *P* | R | *P* |
| Baseline Evaluation | UPDRSIII | 0.101 | **0.037** | 0.140 | **0.004**^*^ | -0.007 | 0.877 | 0.027 | 0.574 | 0.120 | **0.013** | 0.055 | 0.253 | -0.009 | 0.846 |
|  | MoCA | -0.016 | 0.748 | -0.017 | 0.728 | 0.066 | 0.173 | 0.017 | 0.725 | -0.042 | 0.381 | -0.014 | 0.774 | -0.090 | 0.063 |
|  | ESS | -0.034 | 0.482 | 0.022 | 0.652 | -0.072 | 0.139 | -0.080 | 0.098 | -0.024 | 0.625 | -0.06 | 0.214 | 0.071 | 0.143 |
|  | GDS | 0.032 | 0.511 | -0.033 | 0.492 | -0.003 | 0.943 | 0.013 | 0.789 | -0.011 | 0.828 | -0.022 | 0.654 | 0.012 | 0.804 |
|  | QUIP | 0.030 | 0.541 | 0.076 | 0.117 | 0.114 | **0.018** | 0.044 | 0.364 | 0.022 | 0.656 | 0.030 | 0.535 | -0.124 | **0.010** |
|  | RBDSQ | 0.049 | 0.315 | 0.007 | 0.880 | -0.008 | 0.870 | 0.025 | 0.609 | 0.033 | 0.495 | 0.023 | 0.629 | -0.002 | 0.964 |
|  | STAI | -0.080 | 0.096 | -0.041 | 0.397 | -0.057 | 0.242 | -0.101 | **0.037** | -0.080 | 0.096 | -0.094 | 0.050 | 0.045 | 0.349 |
|  | UPSIT | -0.056 | 0.245 | -0.056 | 0.244 | -0.025 | 0.610 | -0.049 | 0.314 | -0.061 | 0.204 | -0.041 | 0.394 | 0.034 | 0.480 |
|  | SCOPA-AUT | 0.072 | 0.138 | 0.018 | 0.712 | -0.015 | 0.760 | 0.061 | 0.205 | 0.074 | 0.127 | 0.056 | 0.245 | -0.030 | 0.537 |
| Longitudinal Evaluation | UPDRSIII change | 0.057 | 0.261 | 0.049 | 0.335 | 0.128 | **0.011** | 0.115 | **0.023** | 0.054 | 0.286 | 0.087 | 0.083 | -0.104 | **0.039** |
|  | MoCA change | -0.111 | **0.031** | -0.047 | 0.363 | -0.026 | 0.617 | -0.081 | 0.116 | -0.070 | 0.174 | -0.045 | 0.384 | 0.049 | 0.349 |
|  | ESS change | 0.083 | 0.108 | 0.084 | 0.103 | 0.024 | 0.636 | 0.047 | 0.357 | 0.072 | 0.162 | 0.040 | 0.434 | -0.030 | 0.567 |
|  | GDS change | -0.011 | 0.831 | -0.091 | 0.077 | -0.019 | 0.716 | -0.007 | 0.891 | -0.049 | 0.340 | -0.042 | 0.412 | -0.015 | 0.768 |
|  | QUIP change | -0.094 | 0.068 | -0.141 | **0.006**^*^ | -0.140 | **0.006**^*^ | -0.110 | **0.033** | -0.098 | 0.058 | -0.103 | **0.045** | 0.123 | **0.017** |
|  | RBDSQ change | 0.014 | 0.779 | 0.005 | 0.926 | -0.012 | 0.818 | 0.002 | 0.974 | 0.008 | 0.874 | -0.012 | 0.813 | 0.000 | 0.995 |
|  | STAI change | 0.001 | 0.987 | -0.021 | 0.680 | 0.113 | **0.029** | 0.065 | 0.208 | -0.027 | 0.600 | 0.020 | 0.704 | -0.093 | 0.072 |
|  | UPSIT change | 0.012 | 0.945 | 0.004 | 0.983 | -0.056 | 0.749 | 0.015 | 0.930 | 0.049 | 0.782 | 0.096 | 0.581 | -0.002 | 0.991 |
|  | SCOPA-AUT change | -0.005 | 0.919 | -0.057 | 0.273 | -0.026 | 0.617 | 0.003 | 0.952 | -0.031 | 0.546 | -0.021 | 0.679 | 0.004 | 0.936 |

Abbreviations: PD, Parkinson’s disease; UPDRS III, Unified Parkinson’s Disease Rating Scale part III; MoCA, Montreal Cognitive Assessment; ESS, Epworth Sleepiness Scale; GDS, Geriatric Depression Scale; QUIP, Questionnaire for Impulsive-Compulsive Disorders; RBDSQ, Rapid Eye Movement Behavior Disorder Screening Questionnaire; SCOPA-AUT, Scales for Outcomes in Parkinson’s disease - Autonomic; STAI, State-Trait Anxiety Inventory; UPSIT, University of Pennsylvania Smell Identification Test; NLR, neutrophil-to-lymphocyte ratio; PLR, platelet-to-lymphocyte ratio; MLR, monocyte-to-lymphocyte ratio; SII, systemic immune-inflammation index; SIRI, systemic inflammation response index; AISI, aggregate index of systemic inflammation; HALP, hemoglobin, albumin, lymphocyte, and platelet. Spearman correlation analysis was used for analysis. Bold values indicate significant differences among groups (*p*<0.05). * *p*<0.007 was taken as cutoff value for significance after multiple correction.

**Supplementary Table 4**. **Associations between peripheral inflammatory markers and CSF biomarkers**

| **Peripheral inflammatory markers** | **Aβ42 (pg/ml)** |  | **pTau**  **(pg/ml)** |  | **tTau**  **(pg/ml)** |  | **α-syn (pg/ml)** |  | **NfL**  **(pg/ml)** |  | **GFAP (ng/ml)** |  |
| --- | --- | --- | --- | --- | --- | --- | --- | --- | --- | --- | --- | --- |
|  | R | *P* | R | *P* | R | *P* | R | *P* | R | *P* | R | *P* |
| NLR | 0.028 | 0.670 | 0.025 | 0.700 | 0.042 | 0.524 | -0.030 | 0.725 | -0.008 | 0.926 | 0.118 | 0.158 |
| MLR | 0.008 | 0.906 | 0.070 | 0.289 | 0.079 | 0.234 | 0.050 | 0.548 | 0.036 | 0.666 | 0.108 | 0.197 |
| PLR | 0.102 | 0.123 | 0.011 | 0.871 | 0.009 | 0.886 | 0.106 | 0.205 | -0.008 | 0.926 | 0.110 | 0.188 |
| SII | 0.046 | 0.488 | -0.067 | 0.310 | -0.060 | 0.360 | -0.039 | 0.638 | -0.052 | 0.538 | 0.058 | 0.491 |
| SIRI | -0.018 | 0.781 | -0.017 | 0.801 | -0.004 | 0.951 | -0.072 | 0.389 | -0.011 | 0.891 | 0.069 | 0.412 |
| AISI | 0.008 | 0.901 | -0.077 | 0.242 | -0.072 | 0.277 | -0.061 | 0.463 | -0.041 | 0.627 | 0.035 | 0.677 |
| HALP | -0.103 | 0.119 | -0.029 | 0.668 | -0.025 | 0.702 | -0.107 | 0.201 | 0.025 | 0.769 | -0.087 | 0.299 |

Abbreviations: NLR, neutrophil-to-lymphocyte ratio; MLR, monocyte-to-lymphocyte ratio; PLR, platelet-to-lymphocyte ratio; SII, systemic immune-inflammation index; SIRI, systemic inflammation response index; AISI, aggregate index of systemic inflammation; HALP, hemoglobin, albumin, lymphocyte, and platelet; α-syn, α-synuclein; pTau, phosphorylated tau; tTau ,total tau; Aβ, β-amyloid; NfL, neurofilament light chain; GFAP, glial fibrillary acidic protein. Spearman correlation analysis was used for analysis.

**Supplementary Table 5. Quartile-stratified analysis of peripheral inflammatory markers and CSF biomarker associations in PD**

| **Peripheral inflammatory markers** | **Aβ42 (pg/ml)** |  | **pTau**  **(pg/ml)** |  | **tTau**  **(pg/ml)** |  | **α-syn (pg/ml)** |  | **NfL**  **(pg/ml)** |  | **GFAP (ng/ml)** |  |
| --- | --- | --- | --- | --- | --- | --- | --- | --- | --- | --- | --- | --- |
|  | Beta | *P* | Beta | *P* | Beta | *P* | Beta | *P* | Beta | *P* | Beta | *P* |
| NLR | -0.233 | 0.544 | 0.267 | 0.304 | 0.183 | 0.481 | -0.045 | 0.571 | 0.353 | **0.020** | 0.477 | 0.278 |
| MLR | 0.141 | 0.498 | 0.190 | 0.240 | 0.153 | 0.346 | 0.036 | 0.652 | 0.227 | 0.428 | 0.422 | 0.142 |
| PLR | -0.202 | 0.375 | -0.242 | 0.206 | -0.282 | 0.140 | -0.331 | 0.285 | -0.313 | 0.322 | -0.252 | 0.427 |
| SII | 0.884 | 0.124 | -0.022 | 0.953 | 0.097 | 0.794 | -0.836 | 0.255 | -0.476 | 0.526 | 0.070 | 0.926 |
| SIRI | -0.134 | 0.787 | -0.434 | 0.248 | -0.305 | 0.417 | -2.147 | **0.001^*^** | -1.347 | 0.067 | -1.014 | 0.117 |
| AISI | -0.380 | 0.478 | 0.159 | 0.648 | 0.049 | 0.889 | 1.799 | 0.666 | 0.982 | 0.198 | 0.402 | 0.599 |
| HALP | 0.083 | 0.398 | -0.111 | 0.213 | -0.107 | 0.230 | -0.081 | 0.533 | 0.001 | 0.996 | 0.140 | 0.292 |

Abbreviations: NLR, neutrophil-to-lymphocyte ratio; MLR, monocyte-to-lymphocyte ratio; PLR, platelet-to-lymphocyte ratio; SII, systemic immune-inflammation index; SIRI, systemic inflammation response index; AISI, aggregate index of systemic inflammation; HALP, hemoglobin, albumin, lymphocyte, and platelet; α-syn, α-synuclein; pTau, phosphorylated tau; tTau ,total tau; Aβ, β-amyloid; NfL, neurofilament light chain; GFAP, glial fibrillary acidic protein. Multiple linear regression was used for analysis. The reported β coefficients represent the mean difference in the outcome per unit change in the predictor. Bold values indicate significant differences among groups (*p*<0.05). * *p*<0.007 was taken as cutoff value for significance after multiple correction.

**Supplementary Table 6. Differences in peripheral inflammatory markers among mutation carriers in PD**

| **Peripheral inflammatory markers** | **Non mutation carriers (N=237)** | **LRRK2**  **carriers (N=104)** | **GBA**  **carriers (N=67)** | **SNCA**  **carriers (N=19)** | **PRKN**  **carriers (N=8)** | **ALL patients**  **(N=435)** | ***P*** |
| --- | --- | --- | --- | --- | --- | --- | --- |
| NLR | 2.408  (1.758-3.041) | 2.409  (1.697-2.981) | 2.533  (2.076-3.276) | 2.277  (1.968-3.160) | 2.567  (2.108-2.998) | 2.436  (1.803-3.069) | 0.435 |
| MLR | 0.239  (0.174-0.299) | 0.218  (0.164-0.292) | 0.240  (0.177-0.278) | 0.221  (0.191-0.262) | 0.218  (0.205-0.254) | 0.230  (0.175-0.288) | 0.909 |
| PLR | 153.374  (122.718-190.405) | 135.135  (107.697-168.158) | 149.123  (112.883-185.938) | 144.231  (121.939-183.929) | 130.456  (89.032-173.356) | 148.571  (116.931-181.722) | **0.045*** |
| SII | 560.000  (375.616-750.534) | 453.652  (395.495-706.725) | 612.446  (453.213-754.473) | 641.006  (379.077-995.644) | 462.735  (414.576-581.063) | 547.571  (398.021-746.103) | 0.158 |
| SIRI | 0.842  (0.548-1.249) | 0.806  (0.539-1.224) | 0.976  (0.599-1.305) | 0.799  (0.521-1.360) | 0.809  (0.744-0.96) | 0.836  (0.551-1.258) | 0.844 |
| AISI | 185.467  (118.403-319.979) | 183.426  (114.491-288.019) | 213.006  (128.494-343.096) | 225.204  (120.962-377.15) | 174.111  (142.167-211.383) | 187.560  (120.535-309.914) | 0.721 |
| HALP | 37.154  (29.453-47.514) | 45.614  (33.49-56.392) | 41.497  (32.335-54.399) | 38.263  (27.504-48.360) | 43.581  (27.545-64.610) | 39.579  (31.353-51.323) | **0.010*** |

Abbreviations: NLR, neutrophil-to-lymphocyte ratio; MLR, monocyte-to-lymphocyte ratio; PLR, platelet-to-lymphocyte ratio; SII, systemic immune-inflammation index; SIRI, systemic inflammation response index; AISI, aggregate index of systemic inflammation; HALP, hemoglobin, albumin, lymphocyte, and platelet. Kruskal-Wallis test was used for analysis. Bold values indicate significant differences among groups (*p*<0.05). *p*<0.007 was taken as cutoff value for significance after multiple correction. ***** significant differences between LRRK2 carriers and non-mutation carriers.

**Supplementary Table 7. Peripheral inflammatory marker profiles across PD clusters**

| **Peripheral inflammatory markers** | **Cluster 1** | **Cluster 2** | ***P*** |
| --- | --- | --- | --- |
| NLR | 2.104(1.558-2.645) | 3.517(3.044-4.06) | **<0.001** |
| MLR | 0.202(0.162-0.264) | 0.331(0.273-0.376) | **<0.001** |
| PLR | 140.881(116.798-172.576) | 214.024(177.941-271.277) | **<0.001** |
| SII | 496.117(351.452-616.649) | 990.77(882.143-1247.932) | **<0.001** |
| SIRI | 0.669(0.512-1.007) | 1.583(1.218-2.001) | **<0.001** |
| AISI | 156.894(109.586-221.349) | 475.57(349.583-604.598) | **<0.001** |
| HALP | 40.185(32.975-52.172) | 27.751(21.634-34.468) | **<0.001** |

Abbreviations: NLR, neutrophil-to-lymphocyte ratio; MLR, monocyte-to-lymphocyte ratio; PLR, platelet-to-lymphocyte ratio; SII, systemic immune-inflammation index; SIRI, systemic inflammation response index; AISI, aggregate index of systemic inflammation; HALP, hemoglobin, albumin, lymphocyte, and platelet. Mann-Whitney U test was used for analysis. Bold values indicate significant differences among groups.

**Supplementary Table 8. Peripheral inflammatory marker profiles across PD clusters**

| **CSF biomarker** | **Cluster 1** | **Cluster 2** | ***P*** |
| --- | --- | --- | --- |
| Aβ42, pg/ml | 843.938±357.659 | 915.674±387.142 | 0.149 |
| pTau, pg/ml | 13.790±6.944 | 11.790±5.695 | **0.021** |
| tTau, pg/ml | 158.6(122.9-194.35) | 144.6(112-181.7) | **0.024** |
| α-synuclein, pg/ml | 109(70.775-139.7) | 97.67(72.99-122.1) | 0.130 |
| NfL, pg/ml | 103.9(80.205-135.55) | 69.1(56.2-96.08) | **<0.001** |
| GFAP, ng/ml | 6.96(5.215-8.62) | 4.89(3.67-6.3) | **<0.001** |

Abbreviations: NLR, neutrophil-to-lymphocyte ratio; MLR, monocyte-to-lymphocyte ratio; PLR, platelet-to-lymphocyte ratio; SII, systemic immune-inflammation index; SIRI, systemic inflammation response index; AISI, aggregate index of systemic inflammation; HALP, hemoglobin, albumin, lymphocyte, and platelet. Independent samples t-test and Mann-Whitney U test were used for analysis. Bold values indicate significant differences among groups.
