# Supplementary figures and images for: Peripheral Inflammatory Biomarkers in Parkinson’s Disease: Clinical Correlations and Stratification
Source: Cell Mol Neurobiol. 2026 Mar 8;46:73. doi: 10.1007/s10571-026-01708-8 (PMC13009453; doi:10.1007/s10571-026-01708-8)

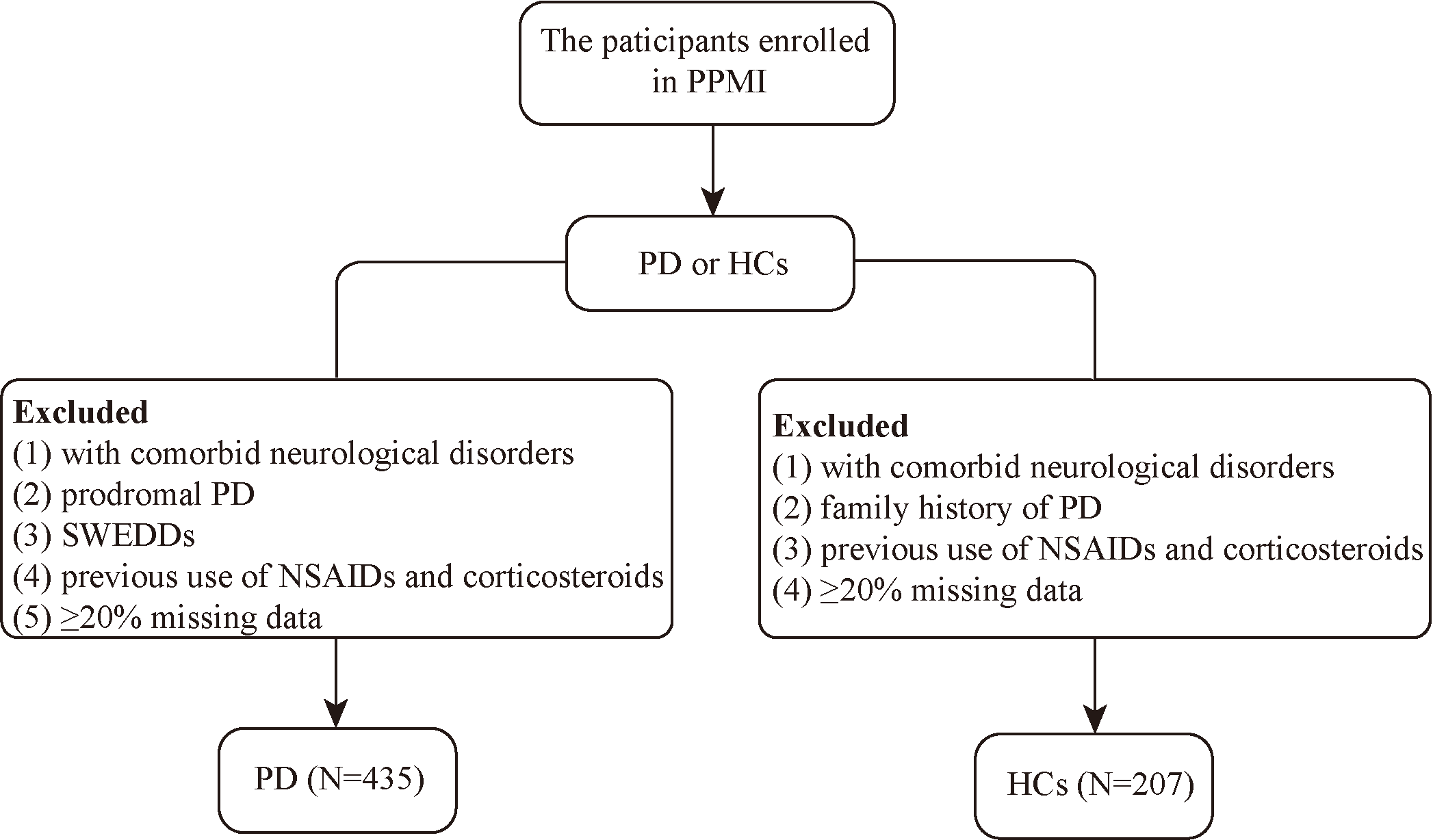

Supplement: Supplementary file 2 — Supplementary Material 2—Study participant inclusion and exclusion flowchart. Abbreviations: PPMI, Parkinson's Progression Markers Initiative; SWEDDs, scans without evidence of dopaminergic deficit; NSAIDs, non-steroidal anti-inflammatory drugs [file 10571_2026_1708_MOESM2_ESM.tif]

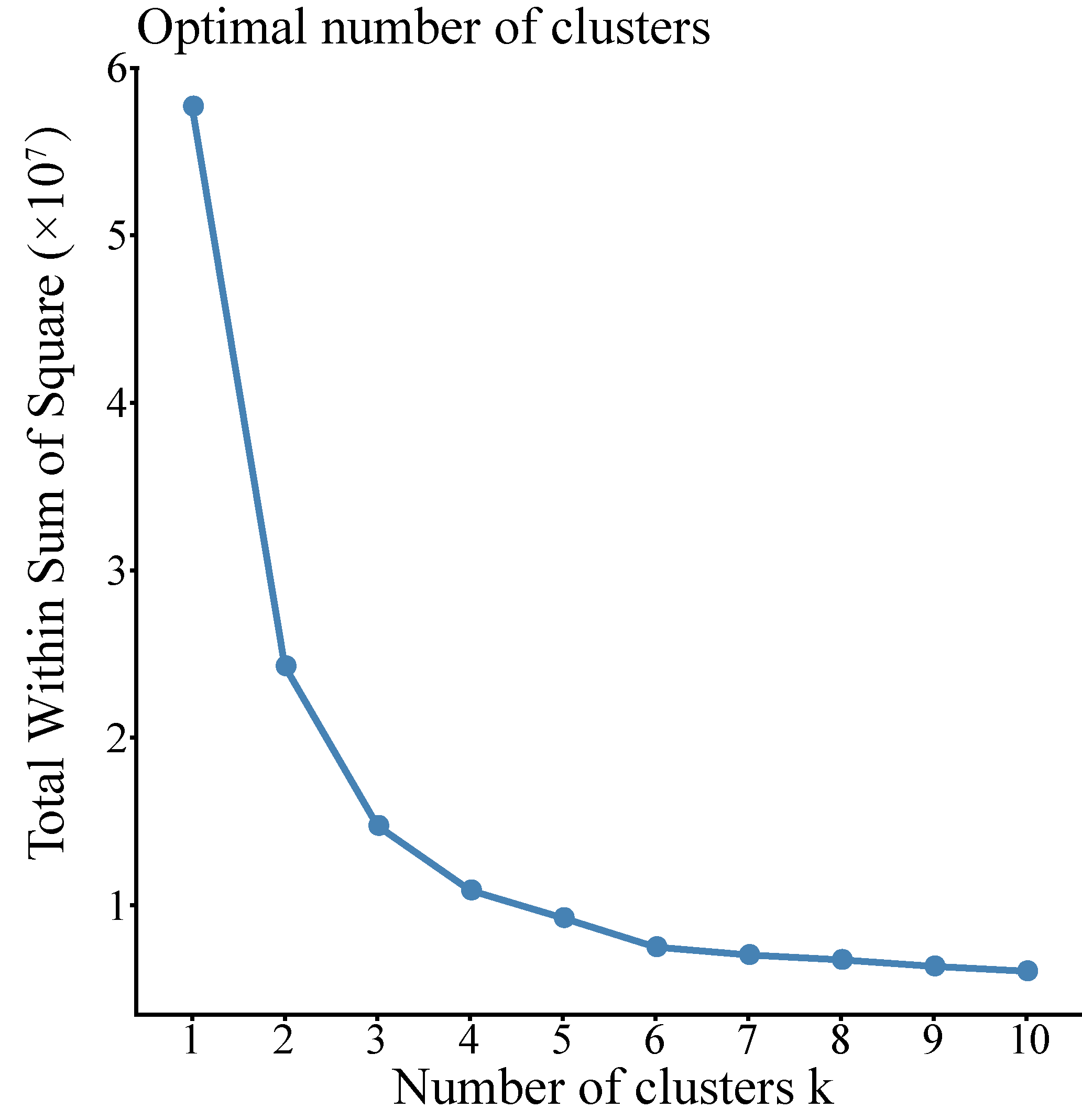

Supplement: Supplementary file 3 — Supplementary Material 3—Selection of optimal cluster number through elbow plots. The optimal cluster number (k=2) was determined via elbow plot analysis of total sum of squares [file 10571_2026_1708_MOESM3_ESM.tif]

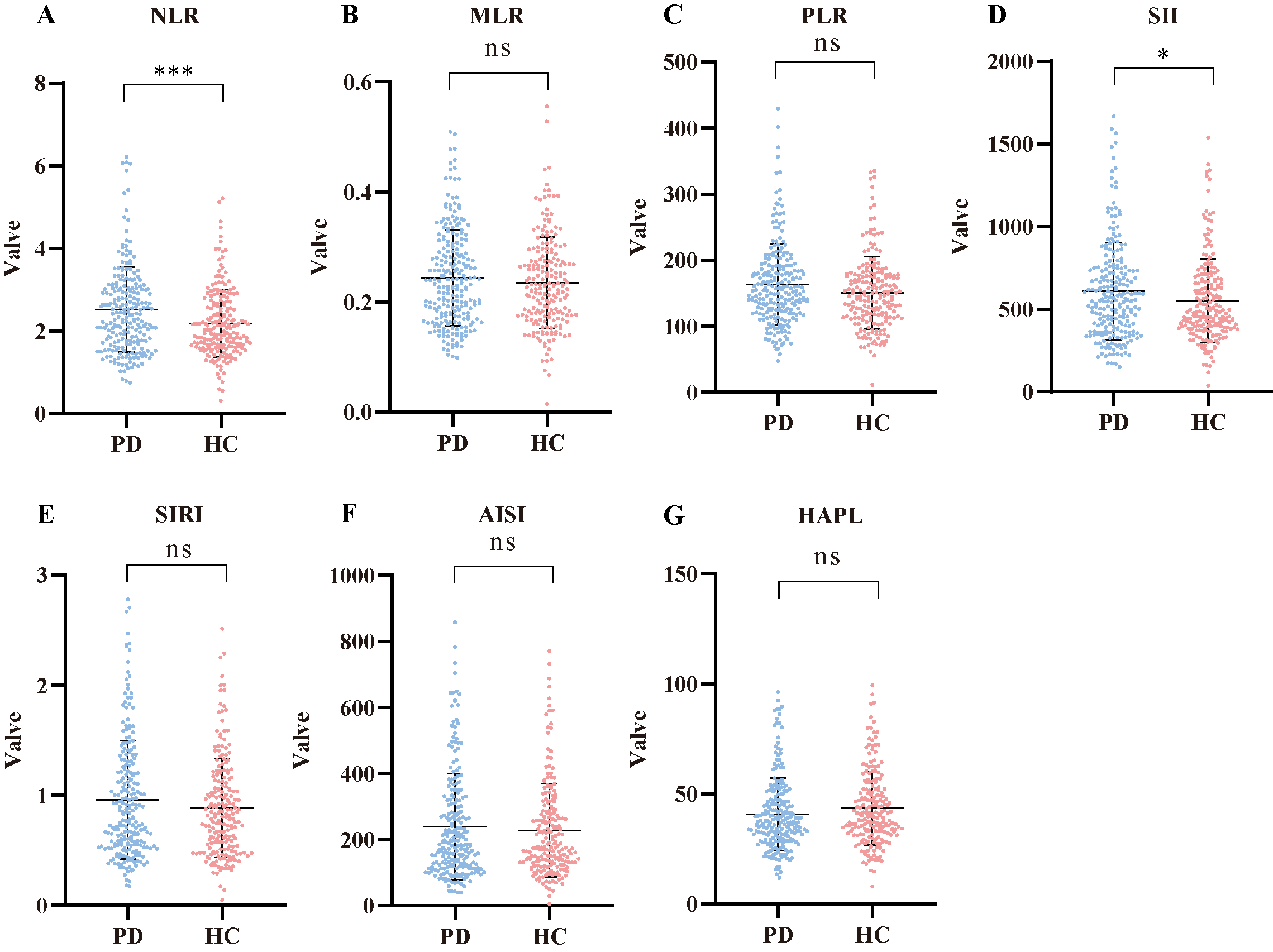

Supplement: Supplementary file 4 — Supplementary Material 4—Comparison of peripheral inflammatory biomarker levels between PD and HC. A NLR; B MLR; C PLR; D SII; E SIRI; F AISI; G HALP. Abbreviations: NLR, neutrophil-to-lymphocyte ratio; PLR, platelet-to-lymphocyte ratio; MLR, monocyte-to-lymphocyte ratio; SII, systemic immune-inflammation index; SIRI, systemic inflammation response index; AISI, aggregate index of systemic inflammation; HALP, hemoglobin, albumin, lymphocyte, and platelet; ns, not significant. * p<0.05; *** p<0.001 [file 10571_2026_1708_MOESM4_ESM.tif]

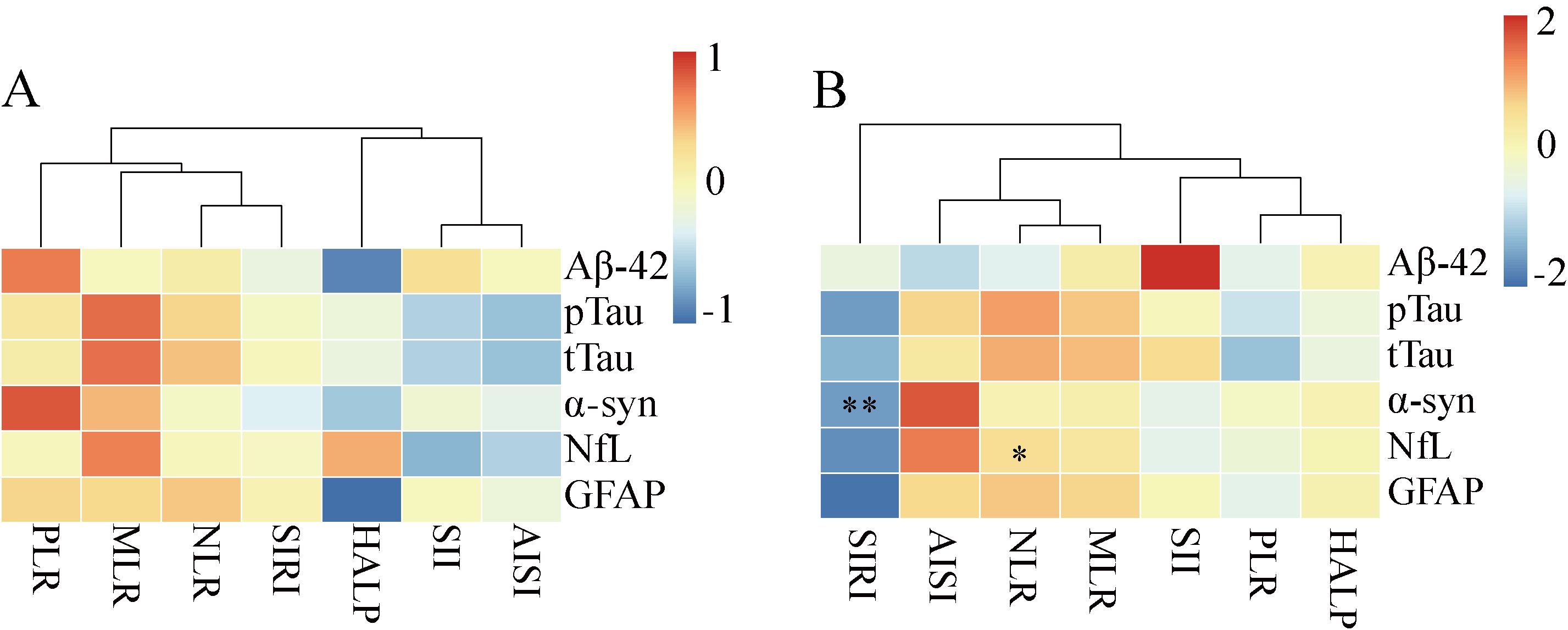

Supplement: Supplementary file 5 — Supplementary Material 5—Associations between peripheral inflammatory biomarkers and CSF biomarkers in PD. A Spearman correlation analysis of peripheral inflammatory markers with CSF biomarker levels. B Multiple linear regression analysis of peripheral inflammatory markers with CSF biomarker levels. The darker the red, the stronger the positive correlation, while the darker the blue, the stronger the negative correlation. * p value passed general significance (p<0.05); ** p passed Bonferroni correction (p<0.007). Abbreviations: NLR, neutrophil-to-lymphocyte ratio; MLR, monocyte-to-lymphocyte ratio; PLR, platelet-to-lymphocyte ratio; SII, systemic immune-inflammation index; SIRI, systemic inflammation response index; AISI, aggregate index of systemic inflammation; HALP, hemoglobin, albumin, lymphocyte, and platelet; α-syn, α-synuclein; pTau, phosphorylated tau; tTau ,total tau; Aβ, β-amyloid; NfL, neurofilament light chain; GFAP, glial fibrillary acidic protein [file 10571_2026_1708_MOESM5_ESM.tif]

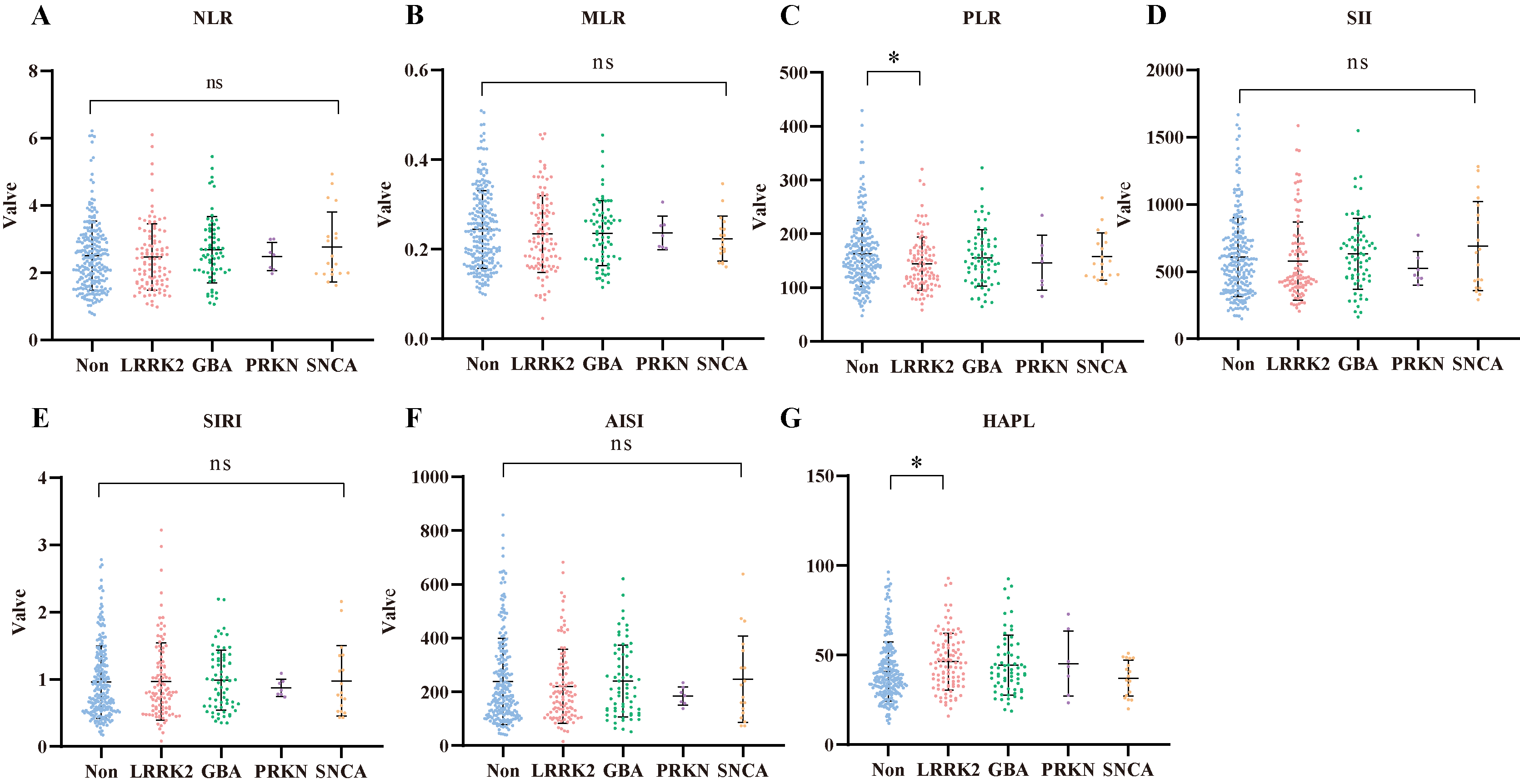

Supplement: Supplementary file 6 — Supplementary Material 6—Comparison of peripheral inflammatory biomarker levels in PD mutation carriers. A NLR; B MLR; C PLR; D SII; E SIRI; F AISI; G HALP. Abbreviations: NLR, neutrophil-to-lymphocyte ratio; MLR, monocyte-to-lymphocyte ratio; PLR, platelet-to-lymphocyte ratio; SII, systemic immune-inflammation index; SIRI, systemic inflammation response index; AISI, aggregate index of systemic inflammation; HALP, hemoglobin, albumin, lymphocyte, and platelet. ns, not significant. * p<0.05 [file 10571_2026_1708_MOESM6_ESM.tif]
